# Subacute thyroiditis during pregnancy: clinical characteristics of seven cases

**DOI:** 10.1530/ETJ-24-0128

**Published:** 2024-10-14

**Authors:** Hideyuki Imai, Natsuko Watanabe, Rei Hirose, Masakazu Koshibu, Masahiro Ichikawa, Akiko Sankoda, Shigenori Hiruma, Nami Suzuki, Masako Matsumoto, Miho Fukushita, Ai Yoshihara, Jaeduk Yoshimura Noh, Kiminori Sugino, Koichi Ito

**Affiliations:** 1 Department of Internal Medicine, Ito Hospital, Tokyo, Japan; 2 Department of Surgery, Ito Hospital, Tokyo, Japan

**Keywords:** fever, hyperthyroidism, hypothyroidism, pregnancy, subacute thyroiditis

## Abstract

**Objective:**

There are few reports of subacute thyroiditis (SAT) during pregnancy. This study aimed to clarify the clinical characteristics of SAT in pregnant patients.

**Methods and results:**

Seven patients diagnosed with SAT during pregnancy at our institution from January 2004 to December 2021 were identified, and their clinical findings were retrospectively examined. At SAT diagnosis, the median age was 34 (range: 31–42) years, the median duration of pregnancy was 5 (4–24) weeks, and all patients had neck pain but no fever. On laboratory examination, median (range) free thyroxine, free triiodothyronine, and C-reactive protein levels were 2.66 (1.14–7.77) ng/dL, 7.1 (3.3–16.1) pg/mL, and 2.22 (0.42–5.79) mg/dL, respectively, and all patients had a hypoechoic lesion of the thyroid gland. Three patients (43%) were treated with steroids, and three patients (43%) received replacement therapy with levothyroxine for hypothyroidism following destructive thyroiditis. There were no pregnancy complications in any of the cases. These seven patients (pregnancy group) were compared with 217 non-pregnant female patients (non-pregnancy group) aged 31 to 42 years who were diagnosed with SAT at our institution from 2016 to 2019. The frequency of body temperatures above 37°C was lower in the pregnancy group than in the non-pregnancy group (0% vs 65%).

**Conclusion:**

Patients who develop SAT during pregnancy may have less fever than non-pregnant patients with SAT. There were no pregnancy complications in the pregnancy group in this study. This suggests that adverse pregnancy outcomes may be avoided by the appropriate management of SAT, including hypothyroidism after destructive thyroiditis.

## Introduction

Thyroid disorders are common in pregnant women. Hypothyroidism is estimated to occur in 4% of pregnancies (0.5% overt and 3.5% subclinical hypothyroidism), and hyperthyroidism occurs in 2.4% of pregnancies (0.6% overt and 1.8% subclinical hyperthyroidism) (1). Graves’ disease (GD) and gestational transient thyrotoxicosis (GTT) are the two most common causes of hyperthyroidism in pregnancy (2). Subacute thyroiditis (SAT) (also called de Quervain’s thyroiditis or granulomatous thyroiditis) is a self-limiting inflammatory disease of the thyroid (3). SAT is one of the causes of thyrotoxicosis, which is accompanied by fever and neck pain due to inflammation. The majority of SAT patients are women between the ages of 30 and 50 years. However, there are few reports of SAT onset during pregnancy, with a total of only eight cases reviewed by Bai *et al*. in 2022, including one of their own cases and seven previously reported cases (4, 5, 6, 7, 8, 9, 10). Hyperthyroidism in SAT during pregnancy may cause maternal and fetal complications (2). Thus, it is important to know in detail the clinical characteristics of patients who develop SAT during pregnancy.

Our institution, Ito Hospital, is a high-volume center specializing in thyroid diseases. Patients diagnosed with SAT during pregnancy at our institution were identified, and their clinical findings were retrospectively examined. First, a case of SAT onset during pregnancy with steroid treatment is presented. Then the clinical features of seven patients who developed SAT during pregnancy are described. Finally, the characteristics of SAT patients during pregnancy are compared to those of non-pregnant SAT patients.

## Methods

### Subjects and methods

First, a case of SAT onset during pregnancy with steroid treatment and levothyroxine (LT4) replacement is presented as case 1. Second, 7 patients diagnosed with SAT during pregnancy who visited our institution from January 2004 to December 2021 were investigated. These patients were the pregnancy group. The diagnosis of SAT was based on clinical features of swelling with pain and tenderness in the thyroid gland, laboratory findings of elevated C-reactive protein (CRP) levels, and a hypoechoic lesion at a painful portion of the thyroid gland confirmed by ultrasonography (11). Clinical characteristics at the time of SAT diagnosis, SAT treatment, and the clinical course in the pregnancy group were examined. Third, 608 female patients under 50 years of age diagnosed with SAT who visited our institution from January 2016 to December 2019 were evaluated. These patients were not pregnant at the time of diagnosis. Patients who had already started steroid treatment at the time of their hospital visit, among others, were excluded. Ultimately, 217 untreated SAT patients aged 31 to 42 years constituted the non-pregnancy group (Supplementary Figure 1, see the section on supplementary materials given at the end of this article). Clinical characteristics were compared between the pregnancy and non-pregnancy groups.

### Assay

Serum free thyroxine (FT4), free triiodothyronine (FT3), and thyrotropin or thyroid stimulating hormone (TSH) levels were measured using electrochemiluminescence immunoassay kits (ECLusys FT4, FT3, and TSH, Roche Diagnostics, Basel, Switzerland). The reference values used in our institution were as follows– FT4: 0.8–1.6 ng/dL, FT3: 2.2–4.3 pg/mL, and TSH: 0.2–4.5 μIU/mL. Thyrotropin receptor antibody (TRAb) levels were measured using TRAb-CT kits (Cosmic, Tokyo, Japan). Levels are expressed in terms of the inhibition index of TRP binding and are presented as percentages. The reference range was defined by the manufacturer as ≤10%. Anti-thyroglobulin antibodies (TgAb) and anti-thyroid peroxidase antibody (TPOAb) were measured using enzyme-linked immunosorbent assay kits (ECLusys Anti-Tg and Anti-TPO, Roche Diagnostics). The reference ranges for TgAb and TPOAb were ≤40 IU/mL and ≤28 IU/mL, respectively.

### Statistical analysis

The data are expressed as medians and ranges for continuous variables and as numbers or percentages for categorical variables. Comparisons of variables between the pregnancy and non-pregnancy groups were conducted using Fisher’s exact test or Wilcoxon rank-sum test. A two-tailed *P*-value <0.05 was considered significant. JMP software (version 14.0.0, SAS Institute Inc., Cary, NC, USA) was used for all statistical analyses.

### Ethics

This retrospective study was approved by the Ethics Committee of Ito Hospital (approval no. 394) and was conducted according to the Declaration of Helsinki and current laws in Japan. The opt-out method was used to give patients the opportunity to refuse to participate in the study.

## Case Presentation

### Case 1

**Figure 1:**
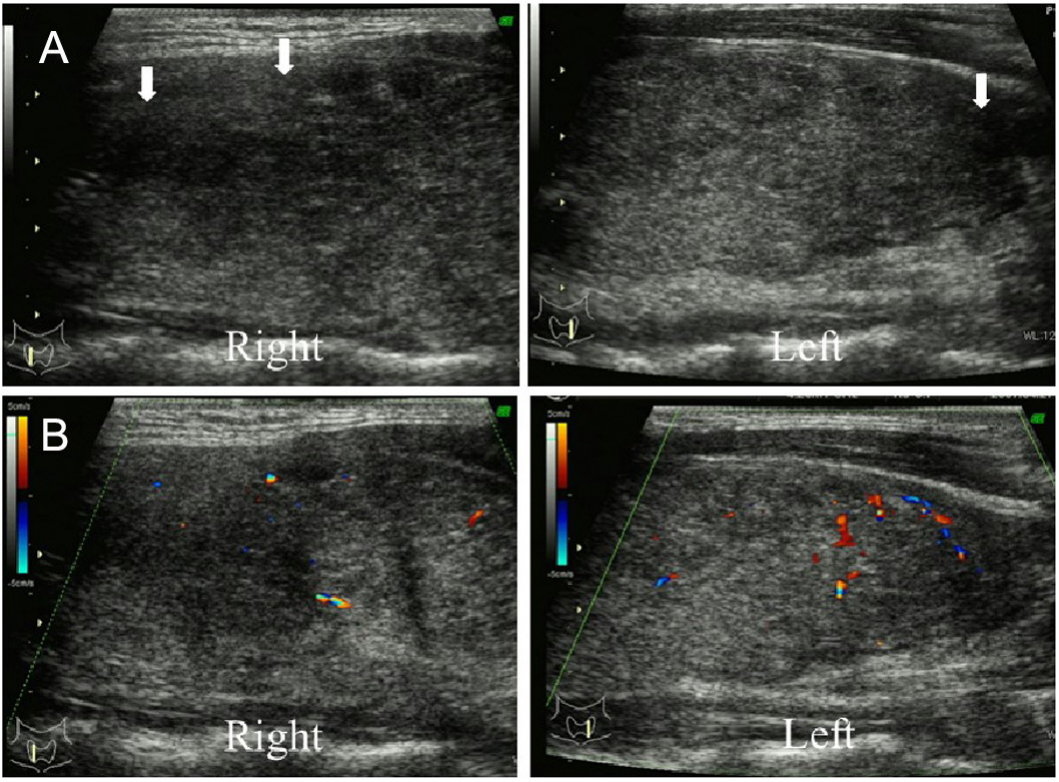
A) Sagittal ultrasonography of the thyroid gland during the initial visit to our hospital in case 1. B) Color-flow Doppler ultrasonography of the thyroid gland in case 1.

**Figure 2:**
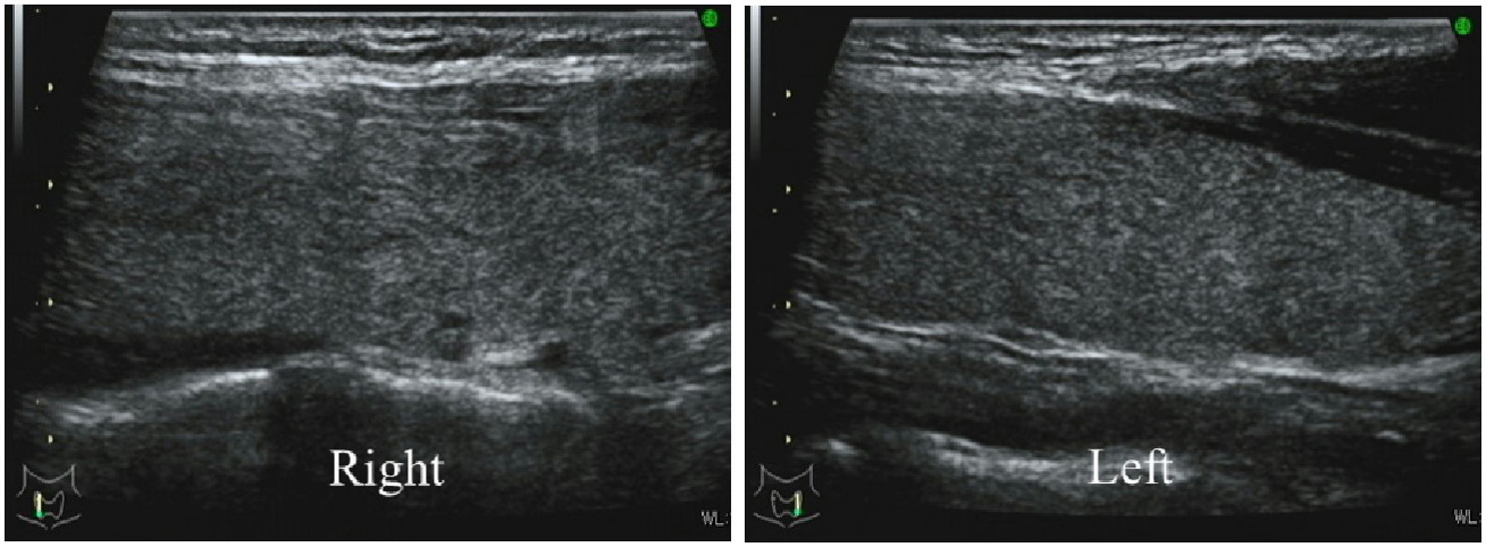
Sagittal ultrasonography of the thyroid gland at 1 month postpartum in case 1.

A 34-year-old Japanese woman, pregnant for the first time, had an uneventful course during the early stages of her pregnancy. From around 23 weeks of pregnancy, the patient experienced pain and swelling on the right side of her neck. At 24 weeks of pregnancy, she visited a local physician. Tenderness was observed in the right thyroid area, leading to suspicion of SAT. She started taking acetaminophen without observing any improvement and made her first visit to our hospital at 25 weeks of pregnancy. There was no medical history or family history of thyroid disease. She presented with tiredness, without symptoms of fever, cough, or sputum. There was no suspicion of a prior viral infection. Cervical palpation showed tenderness in the thyroid gland, not only on the right but also on the left, with creeping thyroiditis. Results of blood tests were as follows– FT4: > 7.77 ng/dL, FT3: 16.1 pg/mL, TSH: < 0.01 μIU/mL, TRAb: 1.9%, TgAb: 21.2 IU/mL, TPOAb: 8.6 IU/mL, and CRP: 5.42 mg/dL (reference range: ≤ 0.30 mg/dL). Thyroid ultrasonography showed hypoechoic lesions in both lobes, corresponding to the region of tenderness. The hypoechoic areas were irregularly shaped with blurred margins and low vascularity on color-flow Doppler (Fig. 1). Based on her symptoms and physical and laboratory findings, she was diagnosed with SAT. Since there was no improvement with acetaminophen, treatment with prednisolone 15 mg per day was started. Sequential laboratory findings and the course of treatment are shown in Table 1. Following the start of prednisolone treatment, her symptoms improved over the course of about 5 days, and CRP levels decreased. At 30 weeks of pregnancy, she developed mild hypothyroidism, and LT4 replacement at 12.5 μg per day was started. At 33 weeks of pregnancy, the dose of prednisolone was reduced to 10 mg per day. At 36 weeks of pregnancy, the dose of prednisolone was further reduced to 5 mg per day, and the dose of LT4 was increased to 25 μg per day. At 38 weeks of pregnancy, there was no recurrence of SAT after discontinuing prednisolone, and she continued to take LT4 25 μg per day until delivery. She gave birth to a male infant at 39 weeks of pregnancy. The baby’s birth weight was 3154 g, and no perinatal complications were observed. Postpartum, LT4 was discontinued. Her thyroid function was normal at one month postpartum. Thyroid ultrasonography showed resolution of the hypoechoic lesions in both lobes (Fig. 2).

**Table 1:** Sequential laboratory findings and the course of treatment in case 1.

	25W	28W	30W	33W	36W	39W^*^	1 month PP
FT4 (ng/dL)	> 7.77	–	0.62	0.89	0.83	–	0.8
FT3 (pg/mL)	16.1	–	1.7	2.1	–	–	2.4
TSH (μIU/mL)	< 0.01	–	0.41	1.15	2.03	–	0.78
CRP (mg/dL)	5.42	1.05	0.71	< 0.10	0.11	–	< 0.10
Prednisolone (mg/day)	15	–	–	10	5		–
LT4 (μg/day)	–	–	12.5	–	25	–	–

CRP, C-reactive protein; FT4, free thyroxine; FT3, free triiodothyronine; LT4, levothyroxine; PP, postpartum; TSH, thyrotropin, W, weeks of pregnancy.

*, delivery.

## Results

### Clinical characteristics of the seven pregnant patients at the time of SAT diagnosis

Clinical characteristics at the time of SAT diagnosis of the seven patients in the pregnancy group are shown in Table 2. At SAT diagnosis, the median age was 34 (range: 31–42) years, the median duration of pregnancy was 5 (4–-24) weeks, five patients (71%) were in the first trimester, and two patients (29%) were in the second trimester. All patients had neck pain, and three patients (43%) had associated creeping. Two patients (29%) had a cough and were therefore suspected of having a prior viral infection. None of the patients had a fever. Thyroid function testing showed median FT4 2.66 (range: 1.14–7.77) ng/dL, median FT3 7.1 (range: 3.3–16.1) pg/mL, and median TSH 0.02 (range: 0.01–0.77) µIU/mL. Thyrotoxicosis was observed in five patients (71%), while two patients (29%) were euthyroid. The median CRP was 2.22 (range: 0.42–5.79) mg/dL, and all patients had high CRP levels. All patients had a hypoechoic lesion of the thyroid gland confirmed by ultrasound. The hypoechoic areas were irregularly shaped with blurred margins and low vascularity on color-flow Doppler.

**Table 2:** Clinical characteristics of the seven pregnant patients at the time of SAT diagnosis. Continuous variables are shown as medians (range). Categorical variables are shown as numbers or percentages.

Year of first visit	Age, years	Weeks of pregnancy	Symptoms	Creeping	FT4 (ng/dL)	FT3 (pg/mL)	TSH (µIU/mL)	CRP (mg/dL)	Hypoechoic lesion
Case 1: 2007	34	24	Neck pain, tiredness	+	> 7.77	16.1	0.01	5.42	+
Case 2: 2007	41	5	Neck pain, cough	−	1.26	3.3	0.77	1.72	+
Case 3: 2014	34	4	Neck pain	+	1.88	4.6	0.02	0.75	+
Case 4: 2013	31	16	Neck pain	−	1.14	3.3	0.43	0.42	+
Case 5: 2016	33	4	Neck pain	−	2.66	7.1	0.02	5.79	+
Case 6: 2021	42	8	Neck pain	+	4.46	13.6	0.01	2.22	+
Case 7: 2020	33	5	Neck pain, cough, sputum	−	3.18	9.1	0.01	5.07	+
Total	34 (31–42)	5 (4–24)	Neck pain: 100% (7/7); cough: 29% (2/7); sputum: 14% (1/7); tiredness: 14% (1/7)	43% (3/7)	2.66 (1.14–7.77)	7.1 (3.3–16.1)	0.02 (<0.01–0.77)	2.22 (0.42–5.79)	100% (7/7)

SAT, subacute thyroiditis, FT4, free thyroxine; FT3, free triiodothyronine; TSH, thyrotropin; CRP, C-reactive protein.

### Treatment and clinical course of the pregnancy group

The treatment and clinical course of the pregnancy group are shown in Table 3. Three patients (43%) were treated with steroids, and one patient (14%) was treated with acetaminophen alone. The three steroid-treated patients were started on 15 mg of prednisolone per day. In one patient, symptoms worsened while on prednisolone 10 mg per day, and the dose was increased to prednisolone 13 mg per day. Three patients (43%) received replacement therapy with LT4 for hypothyroidism following destructive thyroiditis due to SAT. The course of delivery and postpartum thyroid function were confirmed in three patients. Two of these patients had been taking LT4 during pregnancy but discontinued it after delivery. One patient had normal postpartum thyroid function, whereas the other exhibited subclinical hypothyroidism, but no LT4 replacement was required. All infants in these cases were full-term births. One infant had a low birth weight, as did his older brother.

**Table 3:** Treatment and clinical course of the pregnancy group.

	Acute treatment	LT4 replacement (per day, μg)	Delivery time (W)	Newborn weight (g)	Postpartum thyroid function
Case 1	Acetaminophen was taken for 1 week, but symptoms did not improve. Switched to prednisolone 15 mg per day at 25 W, tapered and stopped at 38 W.	12.5–25	39	3154	Normal range
Case 2	Untreated	None	Not provided	Not provided	Not provided
Case 3	Untreated	None	38	2298	Normal range
Case 4	Untreated	None	Not provided	Not provided	Not provided
Case 5	Started prednisolone 15 mg per day at 4 W and tapered. However, symptoms worsened while on prednisolone 10 mg per day. Increased to prednisolone 13 mg per day, tapered and stopped at 16 W.	None	Not provided	Not provided	Not provided
Case 6	Started acetaminophen 800 mg per day, tapered and stopped after 1 month.	75	Not provided	Not provided	Not provided
Case 7	Started prednisolone 15 mg per day at 5 W, tapered and stopped at 14 W.	25–62.5	40	3355	Subclinical hypothyroidism

LT4, Levothyroxine-; W, weeks of pregnancy.

### Comparisons of clinical characteristics between the pregnancy and non-pregnancy groups

Comparisons of clinical characteristics between the pregnancy (seven patients) and non-pregnancy (217 patients) groups are shown in Table 4. There was no significant difference in age between the two groups. The frequency of body temperatures above 37°C was lower in the pregnancy group than in the non-pregnancy group (0% vs 65%). There were no significant differences in FT4, FT3, and TSH between the two groups. Although the frequency of euthyroidism tended to be higher in the pregnancy group than in the non-pregnancy group (29% vs 11%), the difference was not significant. There was no significant difference in CRP levels. Although the frequency of prednisolone treatment tended to be lower in the pregnancy group than in the non-pregnancy group (43% vs 79%), the difference was not significant. Recurrence in patients treated with prednisolone was defined as the presence of symptoms such as tenderness, painful goiter, and CRP elevation. On recurrence, prednisolone was increased or restarted. There were no significant differences in the initial prednisolone dose, frequency of recurrence, total period for prednisolone treatment, or total prednisolone dose between the two groups. The frequency of LT4 replacement was higher in the pregnancy group than in the non-pregnancy group (43% vs 11%).

**Table 4:** Comparisons of clinical characteristics between the pregnancy and non-pregnancy groups. Continuous variables are shown as median (range). Categorical variables are shown as *n* (%).

	Pregnancy group, *n* = 7	Non-pregnancy group, *n* = 217
Age, years	34 (31–42)	37 (31–42)
Body temperature above 37°C	0 (0)^†^	138 (64.5)
FT4 (ng/dL)	2.66 (1.14–7.77)	2.90 (0.95–7.77)
FT3 (pg/mL)	7.1 (3.3–16.1)	7.5 (2.5–23.6)
TSH (µIU/mL)	0.02 (0.01–0.77)	0.01 (0.01–3.93)
Euthyroidism	2 (28.6)	23 (10.6)
CRP (mg/dL)	2.22 (0.42–5.79)	3.50 (0.15–14.0)*
Prednisolone treatment	3 (42.9)	172 (79.3)
Initial prednisolone dose (mg)	15 (15)	15 (5–30)
Recurrence	1 (33.3)	58 (33.7)
Total period for prednisolone treatment (days)	82 (64–96)	70 (7–311)
Total prednisolone dose (mg)	770 (472.5–1120)	602.5 (35–2605)
LT4 replacement	3 (42.9)^†^	24 (11.1)

† *P*-value <0.05; *n=189.

CRP, C-reactive protein, FT4, free thyroxine; FT3, free triiodothyronine; LT4, levothyroxine; TSH, thyrotropin.

## Discussion

SAT is the most common painful thyroid disease with symptoms of hyperthyroidism. Overt hyperthyroidism in pregnancy is associated with maternal and fetal complications, including pre-eclampsia, pregnancy loss, maternal and fetal congestive heart failure, maternal thyroid storm, preterm labor, intrauterine growth retardation, low birth weight, and fetal and/or neonatal hyperthyroidism (2). Inflammation may also cause preterm birth (12). Therefore, pregnant SAT patients need appropriate treatment.

Patients with mild symptomatic SAT should be treated with nonsteroidal anti-inflammatory drugs (NSAIDs) (13). Acetaminophen is sometimes used (14). Steroids should be used instead of NSAIDs when patients fail to respond or present initially with moderate to severe pain and/or thyrotoxic symptoms (13). Sato *et al*. conducted a study to compare NSAIDs and prednisolone for the treatment of SAT, using an initial dose of prednisolone 15 mg per day. The time taken for normalization of thyroid dysfunction was similar in the prednisolone and NSAID groups, but the time taken for the disappearance of initial symptoms was significantly shorter in the prednisolone group (15). Kubota *et al*. showed that an initial dose of 15 mg per day of prednisolone followed by tapering by 5 mg every 2 weeks was an effective therapy for Japanese SAT patients (16). In the present study, there were no pregnancy complications in the seven patients diagnosed with SAT during pregnancy. However, three of the seven patients (43%) in the pregnancy group required steroid treatment. The three steroid-treated patients were started on prednisolone 15 mg per day, and 1 patient recurred. Bai *et al*. reviewed a total of eight cases of SAT onset during pregnancy, including one of their own cases and seven previously reported cases (4, 5, 6, 7, 8, 9, 10). In the previous reports, four cases (50%) were treated with steroids (4, 5, 7, 9). In one of these cases, the pregnancy was terminated because of severe nausea and vomiting, which was partly due to the self-discontinuation of steroids (7). In severe cases, like case 1 of this report, it is considered beneficial to use steroids.

The use of medications during pregnancy may potentially have adverse effects on the fetus. Several previous epidemiological studies have reported an association between corticosteroid use in early pregnancy and the delivery of an infant with an orofacial cleft (17). However, no association was observed in recent studies (18, 19). Exposure to NSAIDs during the last trimester of pregnancy is associated with an increased risk of premature closure of the fetal ductus arteriosus and persistent pulmonary hypertension in the newborn (20, 21). Acetaminophen is considered relatively safe for pregnant women. However, long-term maternal use of acetaminophen has been reported to be associated with attention-deficit/hyperactivity disorder in childhood (22). Even acetaminophen should be used at the lowest effective dose necessary and not indiscriminately (23). The benefits and risks of using any of these medications for pregnant women should be taken into consideration.

SAT patients may develop transient or permanent hypothyroidism after the acute phase (3). It has been reported that 10–15% of SAT patients develop permanent hypothyroidism (24). Maternal overt hypothyroidism increases the risks of pre-eclampsia, gestational diabetes mellitus, preterm birth, caesarean section, and infant intensive care unit admission (25). In the present study, three of the seven patients (43%) in the pregnancy group required LT4 replacement. The frequency of LT4 replacement was higher in the pregnancy group than in the non-pregnancy group. This may be attributed to the willingness to administer LT4 in the pregnancy group to prevent pregnancy complications due to hypothyroidism. In a review of eight cases by Bai *et al*., six cases (75%) also required LT4 replacement (4, 5, 6, 8, 9, 10). Transient hypothyroidism following destructive thyroiditis due to SAT in non-pregnant women does not usually require LT4 treatment, but pregnant patients with SAT need close follow-up and appropriate management after acute treatment.

The Rochester Epidemiology Project reported that the incidence of SAT was 12.1 per 100 000 person-years (py), and higher in females (19.1 per 100 000 py) than in males (4.4 per 100 000 py). The incidence of SAT was the highest in young adulthood (24 per 100 000 py for ages 30–40 years) and middle age (35 per 100 000 py for ages 40–50 years), decreasing with increasing age (26). However, there are few reports of SAT onset during pregnancy. Our institution, Ito Hospital, is a hospital specializing in thyroid diseases. In this study, only seven patients were diagnosed with SAT during pregnancy in the 18 years from January 2004 to December 2021. In the 4 years from January 2016 to December 2019, 608 female patients under the age of 50 years were diagnosed with SAT, but none of these patients was pregnant. Although this study has the limitation of being a retrospective, single-center study in a hospital specializing in thyroid diseases, it suggests that the onset of SAT during pregnancy may be rare.

Susceptibility to SAT has been considered to be associated with the presence of certain types of human leukocyte antigens (HLAs). In particular, a significant correlation between SAT and HLA-B*35 has been confirmed in several studies (11). Although a viral infection has often been implicated as the cause of SAT, the etiology is still unclear (4). A transient immune response is observed in SAT. The thyroid gland is infiltrated by T cells, and they are sensitized to antigens in thyroid extracts during the active phase of SAT (27). It has been reported that Th2 cell activity is increased and B cell activity is decreased in pregnancy (28). Therefore, the course of autoimmune diseases may change during pregnancy. For example, the symptoms of GD or rheumatoid arthritis tend to improve with immune tolerance (2, 29). Similarly, changes in the immune response during pregnancy may explain the low incidence of SAT.

In the present study, which compared the pregnancy and non-pregnancy groups of SAT patients, none of the patients in the pregnancy group had fever, and the frequency of fever above 37°C was lower in the pregnancy group than in the non-pregnancy group. In a review of eight cases by Bai *et al*. only two (25%) had fever. However, in both cases, the fever was below 38°C. Patients who develop SAT during pregnancy may have less fever than non-pregnant patients with SAT. Regarding thyroid function, two out of the seven patients (29%) in the pregnancy group were euthyroid. These patients had no laboratory evidence of thyrotoxicosis but exhibited clinical characteristics and ultrasound findings consistent with SAT. Neck pain might have preceded the appearance of thyrotoxicosis, with abnormal thyroid function developing at a later stage. The frequency of euthyroidism tended to be higher in the pregnancy group compared to the non-pregnancy group (29% vs 11%), although the difference was not significant. Patients who develop SAT during pregnancy may have a lower incidence of fever and may not always exhibit thyrotoxicosis. Therefore, it is important to perform ultrasound in pregnant women presenting with pain in the thyroid region, even in the absence of these findings, to confirm SAT.

The frequency of prednisolone treatment was lower in the pregnancy group than in the non-pregnancy group (43% vs 79%), although the difference was not significant. This may be partly due to the lower incidence of fever in the pregnancy group and partly due to physicians’ reluctance to initiate steroid treatment in pregnant women because of concerns about adverse effects. There were no significant differences in CRP levels, frequency of recurrence, total period for prednisolone treatment, or total prednisolone dose between the two groups. Although there was no fever in the pregnancy group, the severity can be said to be equivalent to that of the non-pregnancy group. SAT is considered a self-limiting inflammatory disease of the thyroid, but in severe cases, it may be advisable to use steroids, similar to the approach in non-pregnant cases. In the present study, there were no pregnancy complications in the pregnancy group. This suggests that adverse pregnancy outcomes may be avoided by the appropriate management of SAT, including hypothyroidism after destructive thyroiditis. Because of the small number of cases, it is necessary to evaluate more cases of SAT during pregnancy in the future.

There are several limitations to this study. This was a retrospective study, and there was no standardized treatment protocol for SAT. Treatment decisions were left to the discretion of individual physicians, resulting in a lack of consistency. Particularly in the pregnancy group, the patients’ pregnancies likely influenced the administration of steroids and LT4. Furthermore, reference values for thyroid function and CRP levels have been reported to change during pregnancy (2). Plasma CRP levels are increased in pregnant women compared to non-pregnant women (30). These changes should have been taken into account in this study.

In conclusion, the onset of SAT during pregnancy is very rare. Patients who develop SAT during pregnancy may have less fever than non-pregnant patients with SAT. Nearly half of the pregnancy group received levothyroxine replacement therapy for hypothyroidism after destructive thyroiditis, and appropriate management of SAT, including hypothyroidism after destructive thyroiditis, to avoid adverse pregnancy outcomes, would be warranted.

## Supplementary Materials

Supplementary Figure 1

## Declaration of interest

The authors declare that there is no conflict of interest that could be perceived as prejudicing the impartiality of the research reported.

## Funding

This research did not receive any specific grant from any funding agency in the public, commercial, or not-for-profit sector.
