# Supplementary material for: Subacute thyroiditis during pregnancy: clinical characteristics of seven cases
Source: Eur Thyroid J. 2024 Oct 14;13(5):e240128. doi: 10.1530/ETJ-24-0128 (PMC11558916; doi:10.1530/ETJ-24-0128)
Supplement: Supplementary Figure 1 [file supplementary_figure_1.pdf]

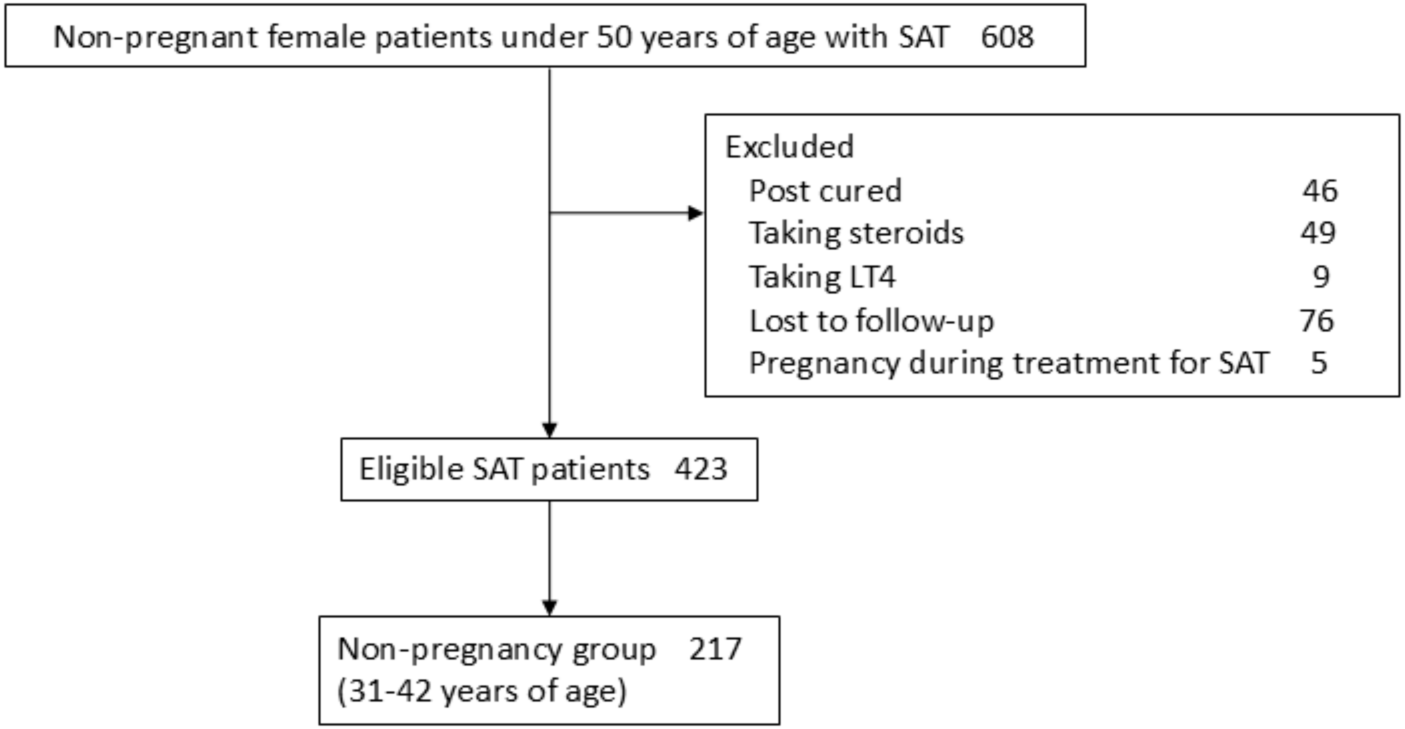

Supplementary Fig. 1 Flow chart showing the inclusion process for the non-pregnancy group. After excluding 391 patients, 217 were analyzed. SAT: subacute thyroiditis, LT4: levothyroxine
